# Optimizing Oral Vitamin C Supplementation: Addressing Pharmacokinetic Challenges with Nutraceutical Formulation Approaches—A Mini Review

**DOI:** 10.3390/pharmaceutics17111458

**Published:** 2025-11-11

**Authors:** Tejal Dhotre, Shefali Thanawala, Rajat Shah

**Affiliations:** Nutriventia Private Limited., Mumbai 400069, India

**Keywords:** ascorbic acid, pharmacokinetic, plasma exposure, renal excretion, sustained-release, systemic retention

## Abstract

Vitamin C, a water-soluble micronutrient, is one of the most widely used dietary supplements pertaining to its vital role in maintaining overall human health, particularly through its potent antioxidant and immune-supportive functions. This mini review summarizes key pharmacokinetic constraints of vitamin C and evaluates formulation strategies aimed at improving its systemic availability. Achieving sustained optimal plasma levels of vitamin C remains challenging due to its dose-dependent absorption, tissue saturation, rapid renal clearance, and short half-life. These pharmacokinetic limitations restrict systemic retention, with high oral doses providing only marginal increases in plasma concentrations and necessitating multiple daily administrations. Conventional vitamin C supplements show efficient absorption only at low to moderate doses, while higher intakes are restricted by transporter saturation and increased renal excretion. Alternative delivery systems such as liposomal encapsulation, esterified derivatives, nano-emulsions, and co-formulations with bioenhancers have been examined; however, evidence for prolonged systemic retention remains inconsistent. The sustained-release formulation of vitamin C shows more reliable outcomes, demonstrating prolonged plasma exposure, higher steady-state concentrations, and potential for improved compliance through reduced dosing frequency. While further robust comparative studies are needed, current evidence suggest that advanced formulation approaches, particularly sustained-release delivery, may help overcome these pharmacokinetic limitations, thereby supporting improved clinical utility of vitamin C supplementation.

## 1. Overview of Vitamin C

Vitamin C, commonly known as L-ascorbic acid, is a vital water-soluble micronutrient involved in a range of biochemical and physiological processes necessary to maintain the body’s overall health. It acts as a powerful antioxidant, supports immune defense, facilitates collagen synthesis, carnitine and catecholamine metabolism, enhances iron absorption, and plays a key role in wound healing [1].

**Figure 1:**
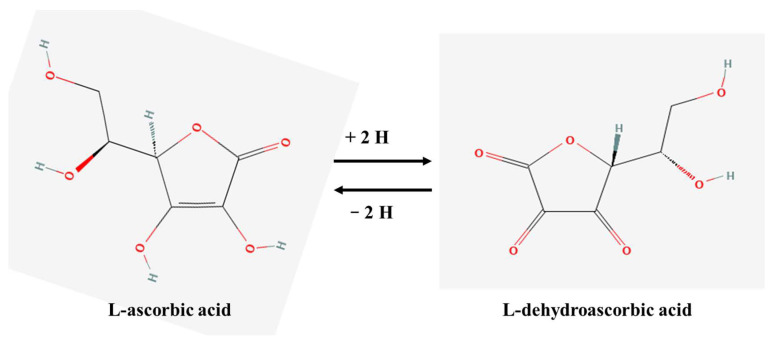
Vitamin C structure in reduced and oxidized forms [2].

Chemically, vitamin C is a six-carbon lactone with enediol structure (C_6_H_8_O_6_; Molar mass = 176.12 g/mol; Melting point = 190–192 °C), which confers high reducing potential and also susceptibility to oxidation under heat, light, and alkaline pH. Its stability is highly sensitive to environmental factors such as air, light, temperature, and the presence of metal ions. In aqueous solutions, L-ascorbic acid undergoes degradation influenced by various parameters, including pH, temperature, and oxidative conditions. In general, ascorbic acid is unstable in aqueous media at room temperature due to its tendency to undergo oxidation to dehydroascorbic acid (DHA). The stability of ascorbic acid improves under acidic conditions, particularly at pH below 2, with maximum stability observed between pH 3 and 6. It exists primarily in two forms—ascorbic acid, the reduced form and dehydroascorbic acid (DHA), an oxidized form (Figure 1) [1,2]. In addition to these, it can transiently exist as the ascorbate free radical, which serves as a key intermediate in redox reactions involving oxidants and underlies the compound’s potent antioxidant activity [1,3].

Unlike most animals, humans lack the enzyme L-gulonolactone oxidase required for endogenous synthesis of vitamin C, making regular dietary intake essential to prevent its deficiency. Natural dietary sources rich in vitamin C include fresh fruits such as citrus, kiwi, and strawberries, as well as vegetables like bell peppers, broccoli, and dark leafy greens [3]. The recommended dietary allowance (RDA) is 90 mg/day for men and 75 mg/day for women, with a tolerable upper intake limit of 2000 mg/day [1,3,4].

Despite widespread use of vitamin C as dietary supplement, achieving optimal plasma and tissue concentrations remains challenging due to its dose-dependent absorption, tissue saturation, and rapid renal clearance. This review examines current literature on vitamin C pharmacokinetics, highlighting challenges such as concentration-dependent uptake and faster urinary excretion, and evaluates the effect of various formulation strategies and technologies on its systemic availability and therapeutic efficacy.

## 2. Vitamin C Pharmacokinetics

Vitamin C pharmacokinetics involve a complex, tightly regulated system influenced by dose, concentration, and tissue-specific factors, marked by dose-dependent absorption, tissue-specific distribution, minimal hepatic metabolism, and regulated excretion. These processes are further influenced by individual physiological and pathological conditions, underscoring the dynamic nature of its systemic availability and therapeutic efficiency [5,6].

At physiological doses, absorption follows saturable active transport kinetics via sodium-dependent vitamin C transporters (SVCT; SVCT1 found in the small intestine and SVCT2 in tissues). In the distal ileum, SVCT1, located on the apical membrane of enterocytes, facilitates sodium-coupled uptake of ascorbate into enterocytes, driven by the Na^+^/K^+^ ATPase gradient. After intracellular accumulation, SVCT2 on the basolateral membrane assists in ascorbate transport into systemic circulation. At higher luminal concentrations, absorption involves passive diffusion, especially of DHA, which is transported via facilitative glucose transporters (GLUT1 and GLUT3). Inside the cell, DHA is rapidly reduced back to ascorbic acid [5,6].

Vitamin C reaches its maximum plasma concentration approximately 2 to 3 h after ingestion [7]. Due to its water-soluble nature, vitamin C is not stored extensively in the body but is preferentially distributed to metabolically active and high-demand tissues, such as the adrenal glands, brain, eyes, and leukocytes. This distribution is extensive but not uniform; vitamin C tissue concentrations can differ by more than 100-fold, governed largely by SVCT2 expression and cellular demand. These homeostatic steady-state concentrations are influenced by dynamic regulation of transport enzymes and are dependent on both systemic levels and tissue needs [4]. Vitamin C further undergoes minimal hepatic metabolism, wherein ascorbate can be reversibly oxidized to DHA, which is reduced intracellularly to maintain redox balance. A fraction is irreversibly degraded to oxalate and other metabolites. Factors such as age, sex, body weight, genetics, and disease states modulate this vitamin C turnover and tissue retention.

Excretion is primarily renal and concentration-dependent. At plasma levels below the renal threshold (~70–100 µmol/L), ascorbate is efficiently reabsorbed in the proximal tubules via SVCT1. Once saturation occurs, excess is eliminated through urine, exhibiting first-order kinetics [5,6].

### Pharmacokinetic Challenges

Despite its biological importance, vitamin C exhibits several pharmacokinetic constraints that might limit its clinical benefits, particularly at supraphysiological doses. A major issue is its non-linear absorption kinetics. At low to moderate oral doses (30–180 mg/day), absorption is efficient, with up to 90% bioavailability. However, beyond 1000 mg/day, fractional absorption declines sharply to less than 50%, primarily due to the saturation of intestinal SVCT1. This leads to a shift from first-order to zero-order kinetics, where further increase in dose no longer proportionally raise systemic availability of ascorbic acid [7].

Another limiting factor is the plasma concentration plateau. Oral doses above 400 mg/day yield only modest increases in plasma ascorbate due to limited absorption and enhanced renal clearance [7]. The kidneys reabsorb ascorbate via SVCT1 in the proximal tubules; however, once plasma levels exceed the renal reabsorption threshold, the kidneys actively eliminate excess ascorbate through urine, reducing its systemic retention. Furthermore, tissue saturation imposes an additional barrier. High plasma ascorbate concentrations, thus, do not necessarily result in elevated intracellular levels, especially in already repleted tissues, where SVCT2-mediated uptake is tightly controlled [5,6,7].

Vitamin C also exhibits a short biological half-life of around 2 h in plasma, again, depending on the dose administered and the physiological status [5]. In healthy individuals, daily intakes of 200–400 mg are sufficient to saturate plasma levels, and any excess is almost entirely excreted through urine [5]. This rapid turnover necessitates multiple daily doses to sustain optimal systemic concentrations, particularly under conditions of oxidative stress or elevated physiological demand.

Additionally, the homeostasis of vitamin C is regulated by a complex interplay of genetic, environmental, and physiological factors. Genetic polymorphisms in transporters such as SVCT1 and SVCT2 can influence intestinal absorption, tissue distribution, and renal reabsorption of ascorbic acid [5,8]. Additionally, lifestyle factors like smoking and poor dietary intake increase oxidative stress, thereby reducing vitamin C status. Chronic diseases such as diabetes, cancer, cardiovascular disease, stroke, diabetes, or sepsis can further disrupt vitamin C homeostasis by substantially altering the overall turnover, leading to suboptimal systemic levels [5].

These pharmacokinetic limitations underscore the need for advanced delivery systems (such as sustained-release formulations, liposomal and encapsulated systems, micronization and nano-emulsion, coformulation with bioenhancers, esterified forms) to overcome traditional absorption bottlenecks, reduce renal losses, and enhance tissue uptake. Such strategies aim to prolong systemic exposure, improve availability of vitamin C in central compartment and improve clinical outcomes, especially in therapeutic settings where vitamin C demand is elevated.

## 3. Nutraceutical Products in the Market—Pharmacokinetic Profile Critique

Conventional vitamin C supplements, such as immediate-release ascorbic acid tablets or capsules, are characterized by rapid absorption, achieving peak plasma concentrations within 2–3 h post-ingestion. However, due to saturation of intestinal SVCT1 and enhanced renal excretion beyond the plasma threshold (~70–90 µmol/L), these formulations exhibit limited systemic retention and tissue saturation, even at high doses. While vitamin C is generally well-tolerated at physiological doses, high doses of vitamin C (>2000 mg/day) may increase the risk of gastrointestinal side effects, including abdominal pain, flatulence, nausea, and diarrhea [4].

Several advanced Vitamin C formulations have been developed to improve absorption, bioavailability, cell or tissue retention, tolerability, and offer broader health benefits (Table 1). However, the robust pharmacokinetic evidence supporting their superiority over standard ascorbic acid remains limited or inconclusive.

One such formulation is calcium ascorbate combined with vitamin C metabolites such as L-threonate. This composition is pH-neutral (non-acidic) and has been clinically demonstrated to remain within immune cells (leukocytes) for up to 24 h, suggesting prolonged cellular retention [10,11]. In a double-blind, crossover study involving 36 healthy adults, the pharmacokinetics of 1000 mg of calcium ascorbate combined with L-threonate and furanone (CA-EC) were compared to those of standard ascorbic acid (AA, 1000 mg) and placebo. Both CA-EC and AA significantly elevated plasma ascorbate levels at 2 h post-ingestion; however, interestingly no significant differences in plasma concentrations were observed between CA-EC and AA across the 24 h monitoring period [11]. These results suggest that while both formulations enhance plasma ascorbate relative to placebo, the inclusion of vitamin C metabolites in the CA-EC formulation does not confer additional systemic bioavailability.

Another pharmacokinetic study (single-dose, randomized, open label, parallel-group) compared the bioavailability of single dose of 500 mg of synthetic vitamin C and neutralized calcium ascorbate in 20 healthy volunteers. Results showed that neutralized calcium ascorbate demonstrated significantly higher bioavailability (128% greater than synthetic vitamin C; *p* < 0.05) based on serum ascorbic acid levels measured over 10 h. These findings suggest that neutralized calcium ascorbate offers superior absorption and potential efficacy compared to conventional synthetic vitamin C. Interestingly, the study measured vitamin C levels only till 10 h post dose, and the fate of administered products remains unevaluated at the end of 24 h [18].

Another formulation that is designed to enhance delivery of vitamin C consists of ascorbic acid combined with lipid metabolites (fatty acids) and citrus bioflavonoids (vitamin C lipid metabolites). In a prospective, randomized, double-blind trial, Pancorbo et al. evaluated the rate of vitamin C absorption in serum after oral administration of four 1000 mg vitamin C formulations—ascorbic acid, calcium ascorbate, ascorbic acid with lipid metabolites, and CA-EC formulation. Authors concluded that the formulation containing ascorbic acid with lipid metabolites resulted in the highest serum vitamin C concentrations, with statistically significant increases at 1, 2, 4, and 6 h compared to calcium ascorbate. In contrast, CA-EC formulation showed limited improvement, with significant increases observed only at 1 and 4 h versus calcium ascorbate. Peak serum levels were reached at 2 h across all formulations, with only slight elevations persisting at 24 h [17]. These findings suggest that the combining of lipid metabolites and bioflavonoids with ascorbic acid may enhance early-phase absorption of vitamin C, with this absorption advantage persisting up to 6 h post-ingestion. However, at 24 h the serum concentrations of the test formulation returned to similar levels as that of other non-lipid-based vitamin C formulations included in the study [17]. Advanced microencapsulation technology is another platform which is used to develop Liposomal-encapsulated forms of L-ascorbic acid, where vitamin C is enclosed within biopolymer or lipid-based carriers, such as liposomes. This technology is intended to protect vitamin C from degradation, enable controlled release, and improve its intestinal absorption. Purpura et al. conducted a randomized, double-blind, placebo-controlled crossover study that evaluated the absorption of liposomal vitamin C compared to standard vitamin C and placebo in 27 healthy adults. Blood samples collected over 24 h post-ingestion of a single oral dose of 500 mg showed that both standard and liposomal vitamin C significantly increased plasma ascorbate levels versus placebo (*p* < 0.001). Notably, the liposomal form demonstrated superior bioavailability in plasma with significantly higher peak concentrations (C_max_: +27%) and area under the curve (AUC_0-24_: +20%) compared to standard vitamin C across 24 h period. These findings indicate that liposomal encapsulation enhances vitamin C absorption [12]. Similarly, another pharmacokinetic study (open-label, randomized, single-dose, two-period crossover study) involving 24 participants compared the oral bioavailability of liposomal vitamin C (1000 mg) with non-liposomal ascorbic acid. The results demonstrated that liposomal formulation demonstrated significantly higher relative bioavailability, with 1.77 times greater total exposure (AUC_0-t_: 55.9 mg·h/dL vs. 31.5 mg·h/dL) and 2.41 times greater rate of absorption (C_max_: 5.2 mg/dL vs. 2.2 mg/dL) than non-liposomal form of vitamin C [19]. These findings highlight the enhanced absorption efficiency of liposomal vitamin C over conventional formulations.

However, although several studies have reported enhanced or superior absorption of liposomal vitamin C, it is important to recognize that vitamin C is inherently highly water-soluble and is generally well-absorbed in any form [19,20,21,22]. To date, no pharmacokinetic study has demonstrated a significant extension of plasma retention or systemic availability beyond 12 h with liposomal formulations. Rather, most available studies have evaluated plasma ascorbate concentrations only up to 6–8 h post-ingestion. In case of liposomal formulations, absorption occurs via the lymphatic system [23]; however, the acidic environment of the stomach renders conventional liposomal vitamin C unstable, resulting in substantial degradation, prior to reaching the absorption sites in the distal small intestine. While few studies suggest improved bioavailability, the elimination kinetics of liposomal vitamin C remain comparable to those of conventional ascorbic acid formulations [24,25,26]. Therefore, to maintain consistent plasma concentrations, requirement of multiple daily doses of liposomal vitamin C is reinforced, consequently resulting in higher concentrations of vitamin C in the gut increasing the possibility of gastrointestinal disturbances [4]. Thus, susceptibility of liposomal vitamin C to degradation in the acidic gastric environment, similar elimination kinetics to conventional vitamin C, and a higher possibility of gastrointestinal side effects at high doses are the limiting factors, restricting its clinical benefits over conventional formulations.

Briefly, the pharmacokinetic evaluation of various vitamin C formulations discussed above underscores two key observations:

- Despite the wide availability of advanced vitamin C formulations and liposomal variants, pharmacokinetic evidence on the benefits offered by these formulations remains limited or inconsistent, with few robust comparative studies to support claims of significantly improved bioavailability or plasma retention up to 6–8 h over conventional ascorbic acid.
- Certain formulations may offer transient advantages such as higher peak plasma concentrations. However, these effects often do not correlate with prolonged systemic availability, and maintaining therapeutic plasma levels may still necessitate multiple daily doses.

## 4. Sustained-Release Delivery Formulations for Optimal Plasma Vitamin C Levels

A depletion–repletion pharmacokinetic study has demonstrated that a daily intake of approximately 200–400 mg of vitamin C is sufficient to saturate plasma levels in healthy individuals. Beyond this intake range, plasma concentrations plateau at a steady-state level of approximately 70–80 μM due to saturation of intestinal absorption and renal reabsorption mechanisms. Notably, after administration of 400 mg vitamin C, between 56% and 80% of the dose was excreted in urine, underscoring the limitations imposed by the body’s homeostatic regulation and rapid renal clearance [7]. These findings suggest that ingestion of multiple smaller doses throughout the day, or administration of a single slow-release formulation, is preferable to a large single dose (>500 mg). Additionally, chronic high-dose consumption may also pose risks such as increased likelihood of calcium oxalate kidney stone formation, especially in individuals with renal dysfunction. Notably, even healthy adults may be at elevated risk when daily intake exceeds the gram dosage. Therefore, to ensure sustained plasma levels and minimize gastrointestinal disturbances, it is recommended that the total daily intake of vitamin C be administered in divided doses or through sustained-release formulations. While vitamin C is generally well-tolerated, daily intake should not exceed the established tolerable upper intake level of two grams for adults [27].

Sustained-release formulations are designed to release the active ingredients gradually over an extended period, thereby maintaining therapeutic plasma concentration and providing a superior bioavailability. Various formulation strategies are used to achieve prolonged release profiles, including diffusion-controlled systems, dissolution-controlled systems, pH-dependent release mechanisms, altered-density systems, osmotic pump technologies, and ion-exchange resins. Among these, matrix-based tablets are the most commonly used platforms for sustained-release formulations. In matrix tablets, the active ingredient is uniformly dispersed within a polymer matrix that modulates the rate of release of active. These can be formulated by either direct compression or granulation method by using a variety of hydrophilic or hydrophobic polymers. These polymers modulate the swelling, dissolution, and erosion of the tablet within the gastrointestinal environment, thereby controlling the diffusion and gradual release of vitamin C over an extended period. Ultimately, the release kinetics of active from matrix systems are primarily governed by the rate and extent of gastric juices penetration, swelling of the polymer, subsequent dissolution and diffusion [28,29].

By modulating the dissolution rate and transit time through the gastrointestinal tract, sustained-release systems aim to extend the absorption window and reduce premature renal elimination [30]. Unlike conventional formulations that produce a typical peak and trough profile with a rapid spike in plasma concentrations followed by rapid renal clearance, the SR formulations provide a gradual release of ascorbic acid, resulting in prolonged systemic exposure and minimizing plasma fluctuations, which generally create a gap in fulfilling the tissue requirement due to inadequate concentrations. The sustained release profile not only reduces the requirement of frequent administration, thereby improving patient adherence; but also reduces gastrointestinal side effects typically observed with high single doses. Additional advantages include reduced total dosage requirements, diminished peak and trough fluctuations, and enhanced availability of the active for continued tissue uptake in a single dose.

**Figure 2:**
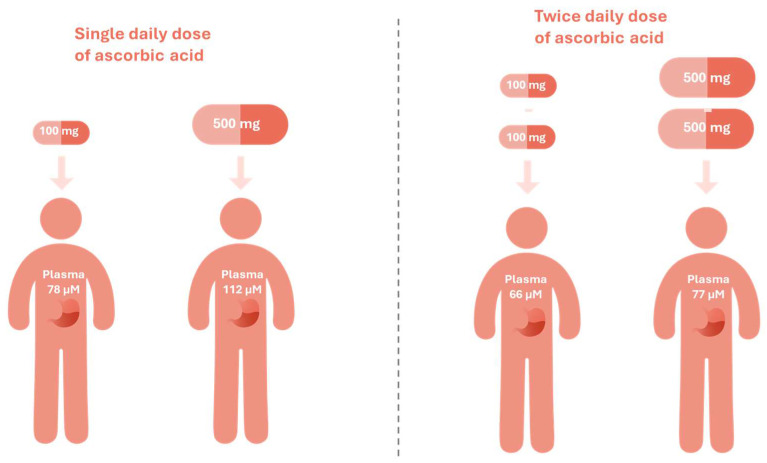
The large-dose fallacy of vitamin C [7,31].

Notably, the slow-release formulations of vitamin C may help surpass the homeostatic saturation threshold and achieve a steady-state plasma concentration of about 250 μM, resulting in a prolonged and thus increased accumulated cellular uptake, as was evidenced in the depletion-repletion study. This landmark study reshaped the understanding of vitamin C pharmacokinetics by resolving the conundrum of the large-dose fallacy. In this study, healthy volunteers were first rendered vitamin C–depleted through dietary restriction. Upon repletion, an initial test dose of 15 mg was administered, followed by gradual increases up to 100 mg and 500 mg. A single 500 mg dose achieved plasma concentrations of 112 μM, representing a ~44% increase compared with 78 μM from a 100 mg dose. Interestingly, when the same doses were administered twice daily (corresponding to 200 mg/day and 1000 mg/day, respectively), plasma concentrations showed minimal further increase (66 μM and 77 μM, respectively) (Figure 2). These findings highlight the saturable nature of vitamin C absorption, wherein higher doses do not substantially increase plasma concentrations [7,31]. Therefore, mimicking the body’s natural, continuous exposure to vitamin C, vitamin C sustained-release formulation might help mitigate the peak and trough in the plasma levels, and rapid renal clearance and thereby help maintain steady-state concentrations and reduce the fluctuation in the tissues as well. Collectively, these features contribute to an improved pharmacokinetic profile and greater clinical efficiency of this water-soluble nutrient.

**Figure 3:**
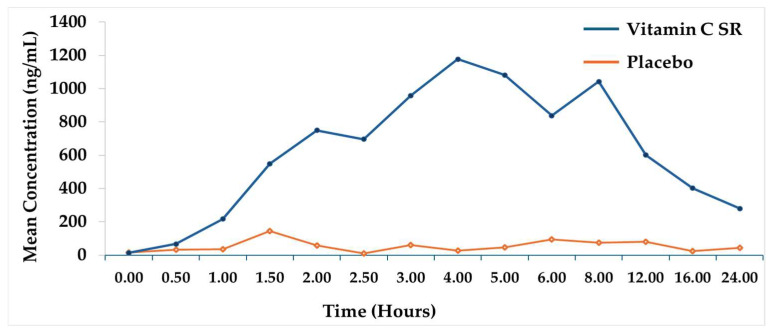
Plasma vitamin C concentration over 24 h after oral administration of sustained-release vitamin C [13].

This postulation is supported by a randomized, placebo-controlled, single-dose pharmacokinetic study that evaluated a novel sustained-release vitamin C formulation (500 mg) in 18 healthy adults under fasting conditions. The sustained-release tablet demonstrated a prolonged plasma ascorbic acid profile, with a mean C_max_ of 1.39 μg/mL and AUC_0–24h_ of 11.72 μg·h/mL, significantly higher than the placebo group. Notably, plasma concentration of vitamin C in the test group was eight times higher than placebo group. The AUC_0–24h_ values indicated that a single 500 mg sustained-release dose maintained elevated vitamin C levels well above the baseline values for the entire 24 h study duration. Furthermore, the mean T_max_ was 4.3 h, in contrast to the 2 h to 3 h observed with immediate-release formulations, which indicates the slow release of the active ingredient. Importantly, plasma concentrations at 12-, 16-, and 24-h post-dose remained substantially above both the baseline and the peak concentration observed in the placebo group [13]. These findings suggest that sustained-release formulations can outwit the absorption saturation and rapid renal clearance limitations associated with conventional vitamin C by prolonging systemic exposure (Figure 3). Such formulations can help maintain plasma vitamin C concentration above the homeostatic saturation level and result in higher steady-state plasma concentration, potentially improving tissue uptake and supporting better compliance through reduced dosing frequency.

Interestingly, contrasting results were reported in a previous single-blind, randomized, placebo-controlled intervention trial that compared the pharmacokinetic profiles of 250 mg immediate-release and 250 mg slow-release vitamin C formulations in 48 healthy active male smokers. This study evaluated plasma retention over the 12 h period; however, no significant difference in the plasma retention between the two formulations was observed [32]. Notably, this study did not extend evaluation of plasma vitamin C to 24 h, in contrast to the above-mentioned pharmacokinetic study of a 500 mg novel sustained-release formulation, which demonstrated prolonged plasma retention with levels approximately eight times higher than the mean peak concentration observed in the placebo group. As compared to the baseline levels, the C_max_ was 85 times higher and vitamin C levels were still 20 times higher at the end of 24 h in the test group [13]. Therefore, it is important to acknowledge that not all sustained-release technologies confer equal benefits, and outcomes may depend primarily on the formulation design. Therefore, development of novel sustained-release delivery systems which can provide actual benefit to the end user remains critical. Future pharmacokinetic studies on different sustained-release vitamin C formulations are warranted to validate their potential in enhancing systemic bioavailability and delivering meaningful clinical benefits.

## 5. Emerging Nano-Formulations and Future Perspectives in Vitamin C Delivery

Recent advancements in nanotechnology have introduced a diverse range of nano-formulation strategies aimed at improving the pharmacokinetic and physicochemical properties of vitamin C. Conventional formulations are often limited by instability, rapid degradation, probably resulting into low therapeutic efficacy; however, nano-based delivery systems such as polymeric, liposomal, micellar, gold, and solid lipid nanoparticles offer significant potential to overcome these challenges. These nanocarriers enhance solubility, protect against oxidative degradation, enable controlled release, and facilitate improved cellular uptake via enhanced permeability and retention effects [15,16].

Nanoformulation platforms such as solid lipid nanoparticles, polymer–lipid hybrid systems, and nanoemulsions have demonstrated considerable potential in enhancing encapsulation efficiency, physicochemical stability, and biocompatibility of delivery systems. The integration of biodegradable polymers, including poly(lactic-co-glycolic acid), chitosan, and alginate, facilitate sustained and controlled release of bioactive compounds, thereby improving therapeutic performance [15,22]. However, the clinical translation of nanoformulated vitamin C systems remains constrained by several inherent challenges. Polymeric nanoparticles often face issues such as particle aggregation, high production costs, and difficulties in getting regulatory approvals, which collectively restrict their large-scale application. Likewise, liposomal carriers are limited by elevated manufacturing expenses, potential fusion or leakage of encapsulated agents, poor solubility, and short half-life, all of which impede their effective evaluation in clinical settings [15].

Currently only a limited number of nano-formulations of vitamin C have reached clinical evaluation. Concentrating on designing hybrid nanocarriers, stimuli-responsive delivery systems, and co-delivery platforms that integrate vitamin C with complementary bioactive compounds or micronutrients to enhance its therapeutic potential might be considered amongst the future approaches.

## 6. Summary

Conventional vitamin C shows efficient absorption at low doses but is limited by saturation kinetics and rapid renal clearance, making it challenging to sustain elevated plasma levels. While alternative formats like liposomal vitamin C and mineral ascorbates have gained popularity, their pharmacokinetic advantages remain inconsistent or limited. Notably, liposomal formulations are prone to gastric degradation and do not significantly extend plasma retention beyond 6–8 h.

In contrast, sustained-release formulations offer a more reliable approach by gradually releasing ascorbic acid, supporting prolonged systemic exposure, steady-state plasma concentrations, and improved compliance with reduced gastrointestinal side effects. Evidence from pharmacokinetic study of sustained-release formulations suggests their potential to overcome conventional limitations and deliver plasma ascorbic acid for prolonged periods, thereby improving systemic exposure, highlighting their promise for long-term clinical benefit. Future research should focus on conducting well-designed comparative pharmacokinetic studies to validate the sustained-release vitamin C formulations over conventional and other formats.

## Figures and Tables

**Table 1:** Vitamin C nutraceutical formulations.

Formulation Type	Delivery Mechanism or Technology
Conventional Ascorbic Acid (Immediate release) (Pure ascorbic acid tablets, capsules, powders)	Rapid dissolution and intestinal absorption via SVCT transporters [5]
Mineral Ascorbates (Sodium ascorbate, Calcium ascorbate, Magnesium ascorbate)	Buffered salts of ascorbic acid- This form is less acidic in the gut resulting in better gastrointestinal tolerability and may allow delivery of large doses with less gastric irritation [9]
Buffered Mineral Ascorbate with metabolite-Enhanced complex (Calcium ascorbate with vitamin C metabolites (dehydroascorbate, calcium threonate, and 4-hydroxy-5-methyl-3(2H)-furanone)	A calcium salt of ascorbic acid is buffered to reduce acidity and improve tolerability. The presence of vitamin C metabolites facilitates its retention in immune cells, allowing for a more sustained presence in the body’s “tissue store” of vitamin C [10,11]
Liposomal Vitamin C (Ascorbic acid encapsulated in phospholipid vesicles)	Encapsulation in liposomes protects vitamin C from degradation in the gastrointestinal tract, enhances intestinal uptake, and improves cellular delivery and retention [12]
Sustained-release (Matrix tablets (hydroxypropyl methylcellulose or lipid-based systems)	By modulating the dissolution rate and transit time through the gastrointestinal tract, sustained-release formulation aims to gradually release the vitamin C in plasma over 8–12 h, maintaining elevated plasma levels for a longer duration. This prolonged release extends the absorption window and mitigates premature renal elimination, thereby enhancing overall systemic exposure [13]
Microencapsulated Vitamin C (Ascorbic acid coated with polymers or lipids)	Micro-encapsulation protects ascorbic acid from oxidation during gastrointestinal transit, and allows controlled release [14]
Nano-formulations (Nanoemulsions, nanoliposomes, solid lipid nanoparticles)	Nanoscale delivery enhances solubility, protects against oxidative degradation, enables controlled release, and facilitates improved cellular uptake via enhanced permeability and retention effects [15,16]
Combination Formulations (Vitamin C with bioflavonoids, vitamin E, zinc, or herbal antioxidants)	Synergistic formulations with other antioxidants or micronutrients may enhance tissue uptake/retention, improve transport into cells, and protect intracellular oxidation of vitamin C that prolongs its retention in cells/tissues [17]

SVCT, sodium-dependent vitamin C transporters.

## Data Availability

Data sharing is not applicable. No new data were created or analyzed in this study.

## Abbreviations

The following abbreviations are used in this manuscript:

DHA	Dehydroascorbic acid
AA	Ascorbic acid
RDA	Recommended dietary allowance
SVCT	Sodium-dependent vitamin C transporters
GLUT	Glucose transporters
CA-EC	Calcium ascorbate combined with L-threonate and furanone
Cmax	Maximum plasma concentration
AUC	Area under curve
